# Mouse guanylate‐binding protein 1 does not mediate antiviral activity against influenza virus *in vitro* or *in vivo*

**DOI:** 10.1111/imcb.12627

**Published:** 2023-02-27

**Authors:** Melkamu B Tessema, Daniel Enosi Tuipulotu, Clare V Oates, Andrew G Brooks, Si Ming Man, Sarah L Londrigan, Patrick C Reading

**Affiliations:** ^1^ Department of Microbiology and Immunology The Peter Doherty Institute for Infection and Immunity, University of Melbourne Melbourne VIC Australia; ^2^ Division of Immunology and Infectious Disease, The John Curtin School of Medical Research The Australian National University Canberra ACT Australia; ^3^ WHO Collaborating Centre for Reference and Research on Influenza, Victorian Infectious Diseases Reference Laboratory The Peter Doherty Institute for Infection and Immunity Melbourne VIC Australia

**Keywords:** GTPase, influenza A virus, innate immunity, interferon‐stimulated gene

## Abstract

Many interferon (IFN)‐stimulated genes are upregulated within host cells following infection with influenza and other viruses. While the antiviral activity of some IFN‐stimulated genes, such as the IFN‐inducible GTPase myxoma resistance (Mx)1 protein 1, has been well defined, less is known regarding the antiviral activities of related IFN‐inducible GTPases of the guanylate‐binding protein (GBP) family, particularly mouse GBPs, where mouse models can be used to assess their antiviral properties *in vivo*. Herein, we demonstrate that mouse GBP1 (mGBP1) was upregulated in a mouse airway epithelial cell line (LA‐4 cells) following pretreatment with mouse IFNα or infection by influenza A virus (IAV). Whereas doxycycline‐inducible expression of mouse Mx1 (mMx1) in LA‐4 cells resulted in reduced susceptibility to IAV infection and reduced viral growth, inducible mGBP1 did not. Moreover, primary cells isolated from mGBP1‐deficient mice (mGBP1^−/−^) showed no difference in susceptibility to IAV and mGBP1^−/−^ macrophages showed no defect in IAV‐induced NLRP3 (NLR family pyrin domain containing 3) inflammasome activation. After intranasal IAV infection, mGBP1^−/−^ mice also showed no differences in virus replication or induction of inflammatory responses in the airways during infection. Thus, using complementary approaches such as mGBP1 overexpression, cells from mGBP1^−/−^ mice and intranasal infection of mGBP1^−/−^ we demonstrate that mGBP1 does not play a major role in modulating IAV infection *in vitro* or *in vivo*.

## Graphical abstract

We have used complementary approaches to demonstrate that neither overexpressed or endogenous mouse guanylate‐binding protein (GBP) 1, an intracellular GTPase, plays a major role in modulating influenza A virus (IAV) infection *in vitro* nor did it modulate IAV in a mouse model of infection. While these studies could be regarded as a “negative” result, they provide clear and important answers to the debate regarding the ability of different GBPs to restrict IAV. They also support the notion that some human and mouse GBPs differ in their biological functions.

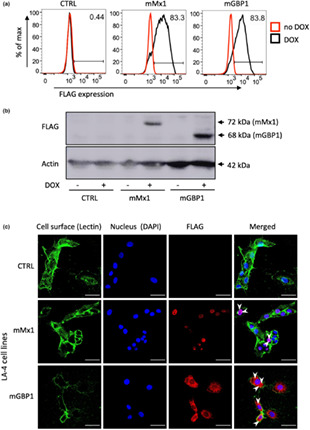

## INTRODUCTION

Since type I interferons (IFNs) were first reported to interfere with viral infections in the 1950s,^1^ research has focused on identifying and understanding IFN‐inducible proteins and their roles in antiviral immunity. Among these IFN‐induced host proteins are the dynamin‐like GTPase family of proteins. Members of this family include myxovirus resistance (Mx) proteins, guanylate‐binding proteins (GBPs) and the immunity‐related GTPases. So far, 7 human and 11 murine GBPs have been identified (reviewed in Man *et al*.^2^) with all human GBPs (hGBPs) localized to chromosome 1 and murine GBPs (mGBPs) mapped to chromosomes 3 and 5. In mice, *Gbp1*, *Gbp2*, *Gbp3*, *Gbp5*, *Gbp7* and one pseudogene are located on chromosome 3, whereas *Gbp4*, *Gbp6*, *Gbp8*, *Gbp9*, *Gbp10*, *Gbp11* and another pseudogene are located on chromosome 5. It is important to note that numbering of GBPs in mice and humans does not necessarily represent orthologs.

It is well established that certain GBPs mediate cell‐autonomous resistance against intracellular bacteria and parasites, either alone or acting in combination (reviewed in Martens and Howard^3^ and MacMicking^4^). It is also well established that Mx proteins (e.g. human MxA and mouse Mx1) can mediate potent antiviral activity against influenza viruses and also against certain other viruses (reviewed in Verhelst *et al*.^5^). While GBPs are known to be upregulated by type I IFNs^6^, ^7^ and some viral infections,^7^, ^8^ the role of particular GBPs in modulating specific viral infections is not so well understood with most studies so far assessing the antiviral activity of different hGBPs *in vitro*. Since Anderson *et al*.^9^ demonstrated that ectopic overexpression of hGBP1 inhibited vesicular stomatitis virus (VSV) and encephalomyocarditis virus, subsequent studies have shown that some hGBPs mediate also antiviral activity against particular viruses, including influenza A virus (IAV).^7^, ^8^, ^10^, ^11^, ^12^, ^13^, ^14^ hGBP1 and hGBP3 have been reported to inhibit IAV replication *in vitro*,^7^ even though the activity of hGBP1 was shown to be antagonized by the viral nonstructural 1 (NS1) protein.^15^ In recent studies, hGBP2 and hGBP5 were shown to interfere with the maturation of the viral envelope in host cells, resulting in antiviral activity against highly pathogenic avian influenza and a range of other enveloped viruses.^14^ hGBP5 was also reported to inhibit IAV replication indirectly by activating the IFN pathway and proinflammatory factors.^16^

In terms of mGBPs, it is well established that mGBP1 plays a critical role in innate immunity against intracellular bacteria such as *Listeria* and *Mycobacteria*,^17^ as well as against protozoa such as *Toxoplasma gondii*.^18^ In terms of antiviral activity, relatively little is currently known regarding the ability of different mGBPs to mediate antiviral activity *in vitro* or *in vivo*. Given that *in vitro* studies have implicated a number of hGBPs in mediating anti‐IAV activity and that mGBP1 has also been reported to inhibit dengue virus *in vitro*,^19^ we investigated whether mGBP1 might also inhibit IAV infection *in vitro* and *in vivo*. Herein, we demonstrate that inducible expression of murine Mx1 (mMx1), but not mGBP1, in a mouse airway epithelial cell line (LA‐4) did not affect susceptibility to IAV infection or virus replication *in vitro*. Furthermore, mice lacking endogenous mGBP1 did not show any differences in susceptibility to IAV infection in terms of virus replication and airway inflammation. Thus, we conclude that mGBP1 is not a major determinant of antiviral activity against IAV *in vitro* or *in vivo*.

## RESULTS

### Induction of murine GBP1 expression in LA‐4 cells by type I IFN or IAV infection

**Figure 1:**
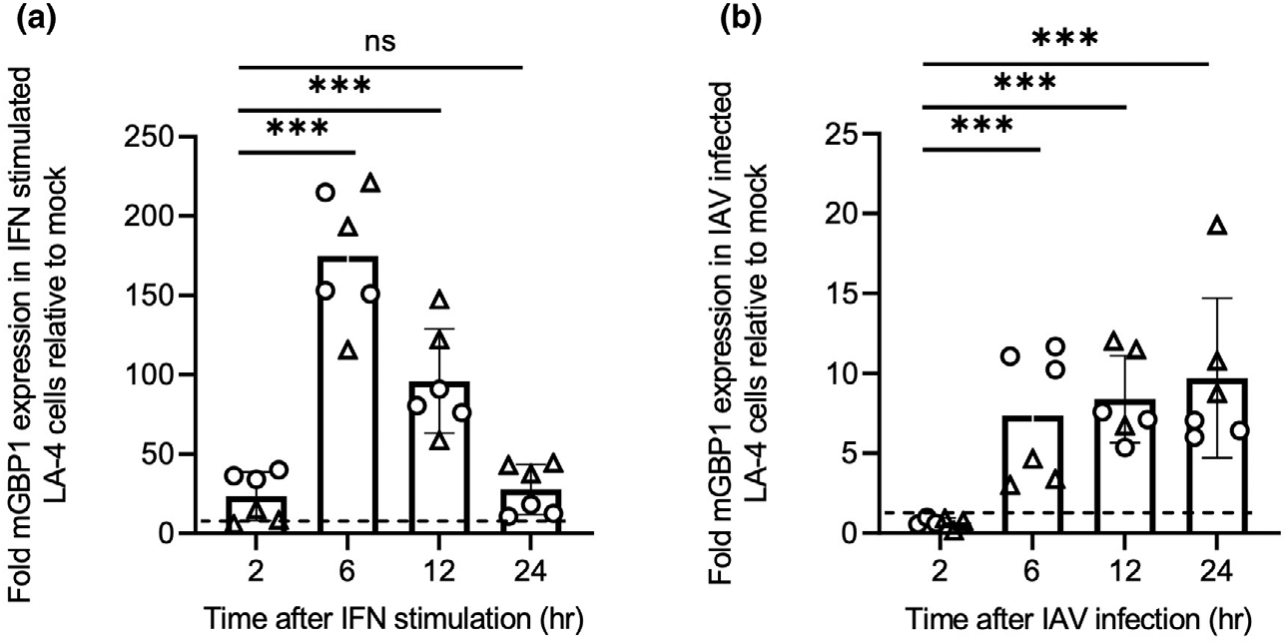
Induction of mGBP1 in LA‐4 cells in response to type I IFN or IAV infection. LA‐4 cells were seeded into 12‐well tissue culture plates (2 × 10^5^ cells/well), cultured overnight and then **(a)** treated with 1000 U mL^−1^ IFNα or with media alone (mock) or **(b)** mock infected or infected with the IAV strain HKx31 (MOI = 5 PFU/cell). At indicated times, RNA was extracted from cells, cDNA synthesized and relative gene expression determined by quantitative real‐time PCR. To assess mGBP1 expression, samples were normalized to mouse GAPDH and then expressed as fold change relative to mock. Data show the mean ± standard deviation expression of triplicate samples pooled from two independent experiments, represented as circles and triangles, respectively. Statistical significance was determined by one‐way ANOVA; ***P < 0.001. The broken horizontal line represents the mGBP1 expression level in the mock. cDNA, complementary DNA; GAPDH, glyceraldehyde 3‐phosphate dehydrogenase; IAV, influenza A virus; IFN, interferon; mGBP1, murine guanylate‐binding protein 1; MOI, multiplicity of infection; PFU, plaque‐forming unit.

First, we investigated induction of mGBP1 expression in a mouse airway epithelial cell line (LA‐4 cells) following treatment with murine type I IFN or infection with the IAV strain HKx31 (H3N2). As seen in Figure 1a, mGBP1 mRNA was rapidly upregulated in response to IFNα with levels significantly higher than mock as early as 2 h following treatment, peaking at 6 h and then gradually declining thereafter. In response to the IAV infection, mGBP1 was upregulated from 6 h and reached a significant level after 12 h and peaked 24 h after infection (Figure 1b). Peak expression levels of mGBP1 were markedly higher following IFN treatment compared with IAV infection (~150‐fold increase at 6 h compared with ~10‐fold increase at 24 h, respectively).

### Generation of LA‐4 cells with inducible expression of mGBP1 and mMx1

**Figure 2:**
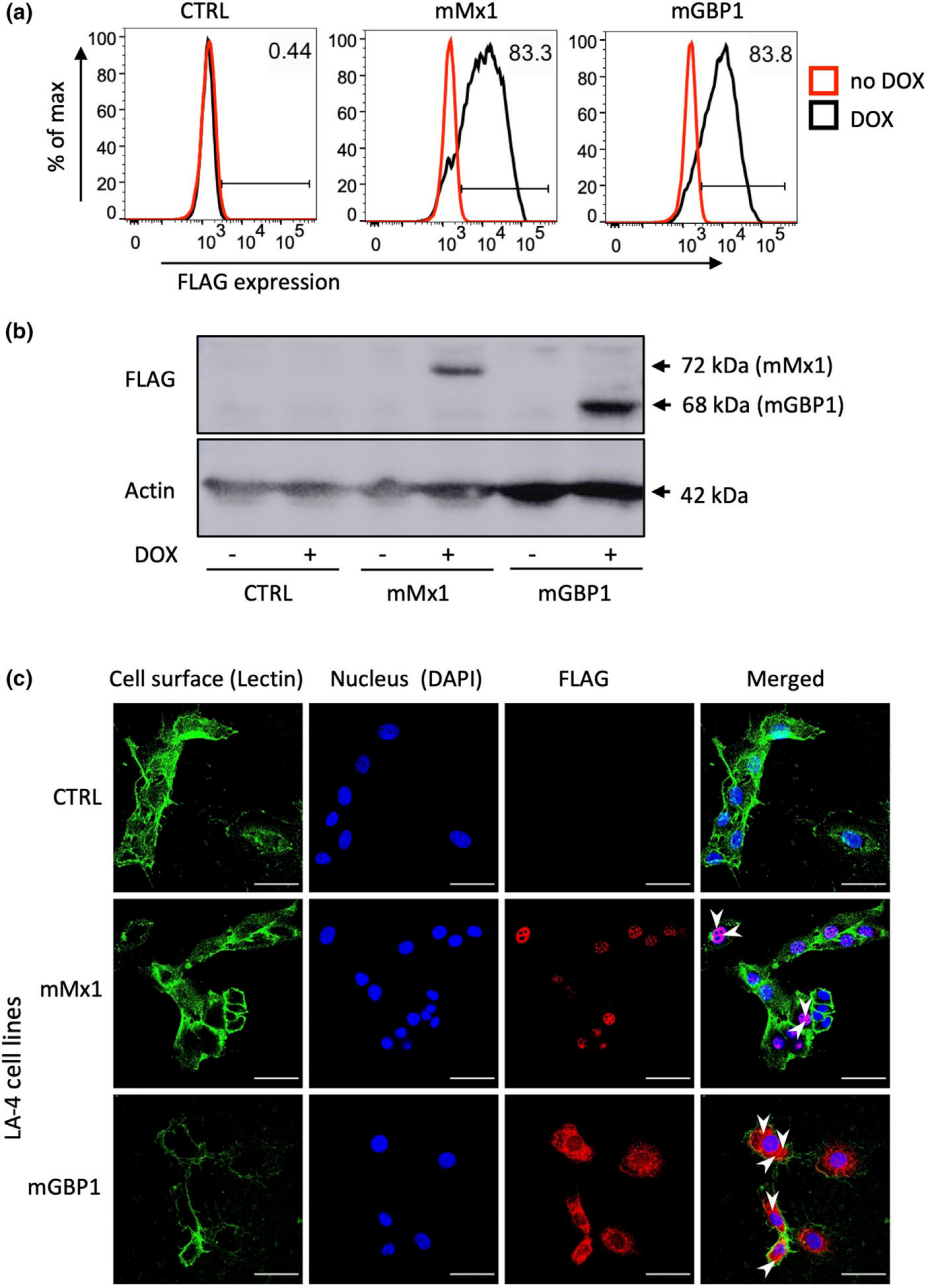
DOX‐inducible expression of mGBP1 and mMx1 in lentivirus‐transduced LA‐4 cells. LA‐4 cells with DOX‐inducible expression of intracellular ovalbumin (CTRL), mMx1 or mGBP1 were seeded at 1 × 10^5^ cells/well into 12‐well tissue culture plates and incubated overnight at 37°C in 5% CO_2_. Media was then supplemented with or without 1 μg mL^−1^ DOX for 24 h. **(a)** After incubation, cells were detached, fixed, permeabilized and stained for intracellular FLAG expression as described in the “Methods” section. Histograms show FLAG expression after incubation in the absence (red) or presence (black) of DOX. Data are representative of three independent experiments. **(b)** Cell lysates were subjected to sodium dodecyl sulfate–polyacrylamide gel electrophoresis, transferred to a polyvinylidene fluoride membrane and probed with anti‐FLAG mAb (upper panel). Membranes were also probed with anti‐β‐actin mAb to confirm similar amounts of cellular protein were loaded for each sample (lower panel). Blot shown has been cropped for presentation. **(c)** Cells were washed, stained for cell‐surface lectin MAL II (lectin) and then fixed, permeabilized and stained for intracellular FLAG and DAPI. Representative images from confocal microscopy show cell‐surface MAL II (green; lectin stain), cell nucleus (blue; DAPI stain), expression of FLAG (red; FLAG tag stain) as well as a merged panel (merged image). The white arrowheads in the merged images indicate mGBP1 expression localizing to the cell cytoplasm and mMx1 expression in the nucleus, by colocalization with DAPI. The scale bar represents 50 μm. CTRL, control; DAPI, 4′,6‐diamidoino‐2‐phenylindole; DOX, doxycycline; FLAG, DYKDDDDK; mAb, monoclonal antibody; mGBP1, murine guanylate‐binding protein 1; mMx1, mouse Mx1.

Although exposure to IFN and IAV can induce mGBP1 expression in LA‐4 cells, we wanted to develop a system to control induction of mGBP1 such that we could examine the impact of high mGBP1 expression prior to IAV infection on subsequent virus replication in LA‐4 cells. Therefore, we used a lentiviral vector system to generate LA‐4 cells with doxycycline (DOX)‐inducible expression of FLAG‐tagged mGBP1. In addition, we generated cells with inducible expression of FLAG‐tagged mMx1, or control cells with inducible expression of an irrelevant protein (cytoplasmic hen egg ovalbumin). The proportion of lentivirus‐transduced (mCherry^+^) LA‐4 cells expressing intracellular FLAG‐tagged proteins was determined using flow cytometry. As seen in Figure 2a, mCherry^+^ LA‐4 cells showed DOX‐inducible expression of FLAG‐tagged mGBP1 and mMx1. Western blot analysis confirmed DOX‐inducible expression of proteins of appropriate size for mGBP1 (68 kDa) and mMx1 (76 kDa; Figure 2b). mGBP1 is usually expressed in the cytoplasm,^20^ whereas mMx1 expression is confined to the nucleus.^21^, ^22^ Confocal microscopy confirmed cytoplasmic expression of DOX‐induced mGBP1 compared with nuclear expression of DOX‐induced mMx1 in LA‐4 cells (Figure 2c).

### DOX‐inducible mMx1, but not mGBP1, mediates antiviral activity against IAV *in vitro*

**Figure 3:**
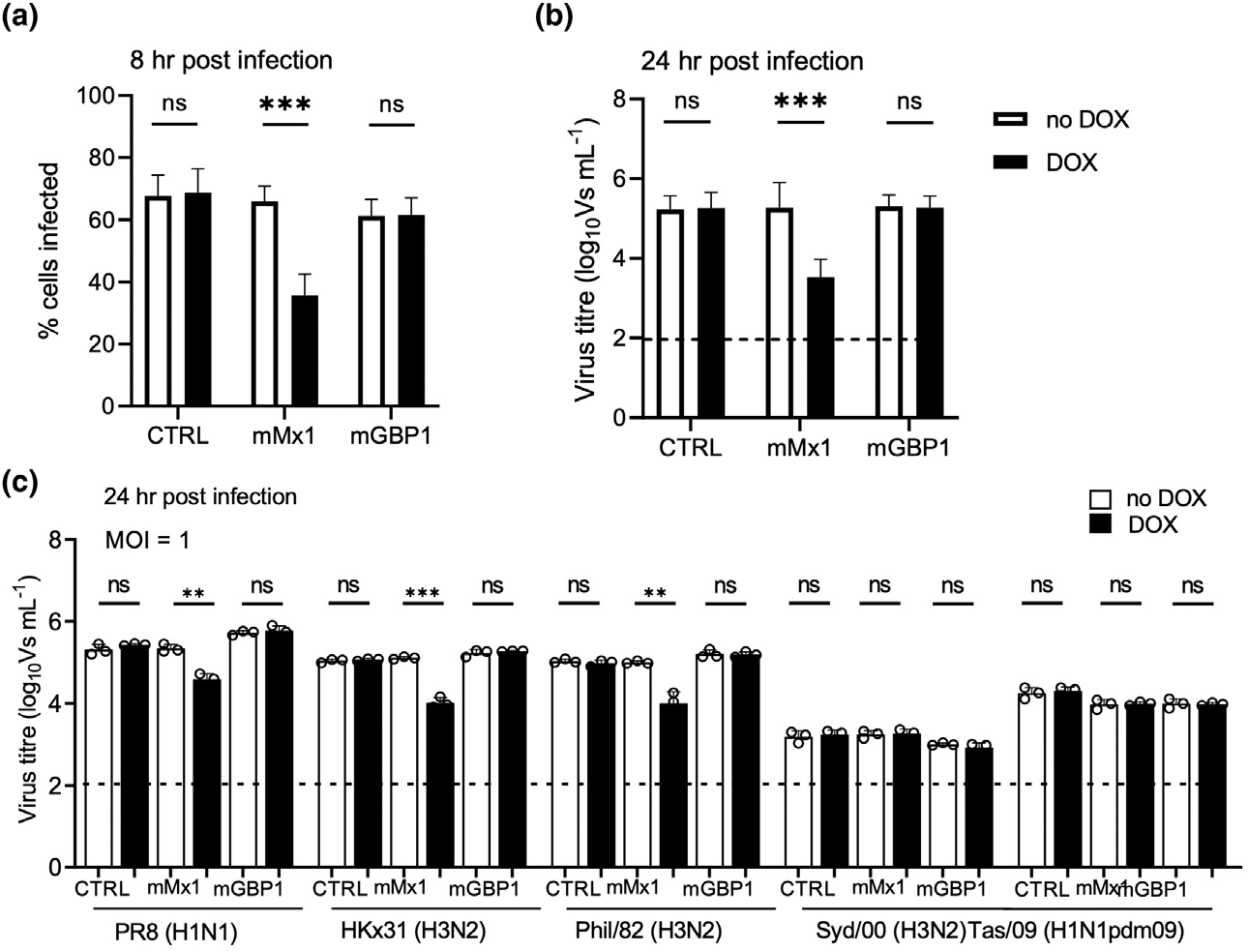
DOX‐inducible mMx1, but not mGBP1, inhibits IAV infection and replication in LA‐4 cells. LA‐4 cells with DOX‐inducible expression of mGBP1 or mMx1, as well as CTRL cells, were seeded at 1 × 10^5^ cells/well into 12‐well tissue culture plates and incubated overnight at 37°C in 5% CO_2_. Media was then supplemented with or without 1 μg mL^−1^ DOX for 24 h. After DOX induction, cells were incubated with **(a, b)** the IAV strain HKx31 (H3N2) at an MOI of 10 or 1 PFU/cell as indicated or **(c)** PR8 (H1N1), HKx31 (H3N2), Phil/82x (H3N2), Syd/00 (H3N2) or Tas/09 (H1N1pdm09) at an MOI of 1 for 60 min at 37°C, washed and cultured at 37°C. **(a)** At 8 h p.i., cells were fixed, permeabilized, stained for intracellular viral NP and analyzed by flow cytometry. (**b, c**) At 24 h p.i., supernatants were removed and clarified, then activated with 2 μg mL^−1^ TPCK‐treated trypsin and titrated on MDCK cells using the VS assay. Data from **a** and **b** show the mean ± standard deviation from three independent experiments. Data from **c** shows the mean ± standard deviation of triplicate samples from one of two independent experiments repeated with similar results. The dashed line in **b** and **c** indicates the detection limit of the VS assay. Statistical significance was determined by the Student's *t*‐test; **P < 0.01; ***P < 0.001. CTRL, control; DOX, doxycycline; IAV, influenza A virus; MDCK, Madin–Darby Canine Kidney; mGBP1, murine guanylate‐binding protein 1; mMx1, mouse Mx1; MOI, multiplicity of infection; NP, nucleoprotein; ns, not significant; PFU, plaque‐forming unit; p.i., postinfection; TPCK, tosylsulfonyl phenylalanyl chloromethyl ketone; VS, ViroSpot.

The anti‐IAV activity of mMx1 is well established (reviewed in^5^); however, much less is known regarding the antiviral activities of related GTPases of the GBP family. Therefore, we investigated whether DOX‐inducible expression of mGBP1 prior to IAV infection modulated (1) the early stages of viral infection, as assessed by detection of newly synthesized viral nucleoprotein (NP) within LA‐4 cells at 8 h after infection; or (2) the later stages of infection involving virus release, by assessing virus infectivity in clarified supernatants 24 h after infection with IAV. As seen in Figure 3a, DOX‐induced mMx1 expression in LA‐4 cells resulted in a significant reduction in the percentage of IAV‐infected cells, whereas LA‐4‐control cells and LA‐4‐GBP1 cells showed similar levels of infection in the presence or absence of DOX. These data confirmed that DOX‐induced expression of mMx1, but not mGBP1, affected one or more steps early in the IAV replication cycle prior to the synthesis of viral NP within infected cells. To investigate whether mGBP1 might impact IAV replication at a late stage in the virus replication cycle, we also examined virus release from LA‐4 cells. Again, while LA‐4‐mMx1 cells showed a > 90% reduction in titers of infectious virus released in the presence of DOX (Figure 3b), LA‐4 control cells and LA‐4‐mGBP1 cells showed no difference in virus titers in the presence or absence of DOX. The potent inhibition of IAV by the FLAG‐tagged mMx1 protein confirms the functionality of recombinant proteins with inducible expression in our system. Together, these data indicate that DOX‐inducible expression of mGBP1 does not significantly impact IAV replication, at least by the approaches used in this study.

Our studies have used the IAV strain HKx31, as this virus replicates efficiently in mice to allow for subsequent studies *in vivo*. mMx1 is reported to differ markedly in its ability to inhibit particular influenza viruses and this is determined by the sequence of the viral NP. Specifically, the NP expressed by human IAV is more resistant to mMx1 than NP from avian strains and mouse‐adapted human IAV.^23^, ^24^ Moreover, it is not known whether other strains of IAV might be sensitive to mGBP1. Therefore, we compared the ability of DOX‐inducible mMx1 and mGBP1 to inhibit a panel of IAV strains, which included mouse‐adapted viruses (PR8, HKx31, Phil/82x), as well as the human strains A/Sydney/203/2000 (Syd/00, H3N2) and A/Tasmania/2004/2009 (Tas/09, H1N1pdm09). In these studies, LA‐4 cells were incubated for 24 h in the presence or absence of DOX, infected with different virus strains and titers of infectious virus in clarified supernatants were determined at 24 h after infection. As seen in Figure 3c, marked differences were noted between strains in the titers of virus released from infected cells, with higher titers generally observed for mouse‐adapted compared with human IAV strains. Despite higher virus titers, the replication of mouse‐adapted viruses expressing internal proteins, including NP, derived from PR8 (i.e. PR8, HKx31, Phil/82x) was potently inhibited by DOX‐inducible mMx1. Of the human viruses, neither the H3N2 nor the H1N1pdm09 strain tested was significantly inhibited by mMx1. Thus, mMx1 potently inhibits IAV expressing NP from mouse‐adapted viruses, but not from human seasonal strains. Of note, DOX‐inducible expression of mGBP1 did not inhibit the growth of any virus strain tested.

### Endogenous mGBP1 does not modulate antiviral activity nor IAV‐induced inflammasome activation *in vitro*

**Figure 4:**
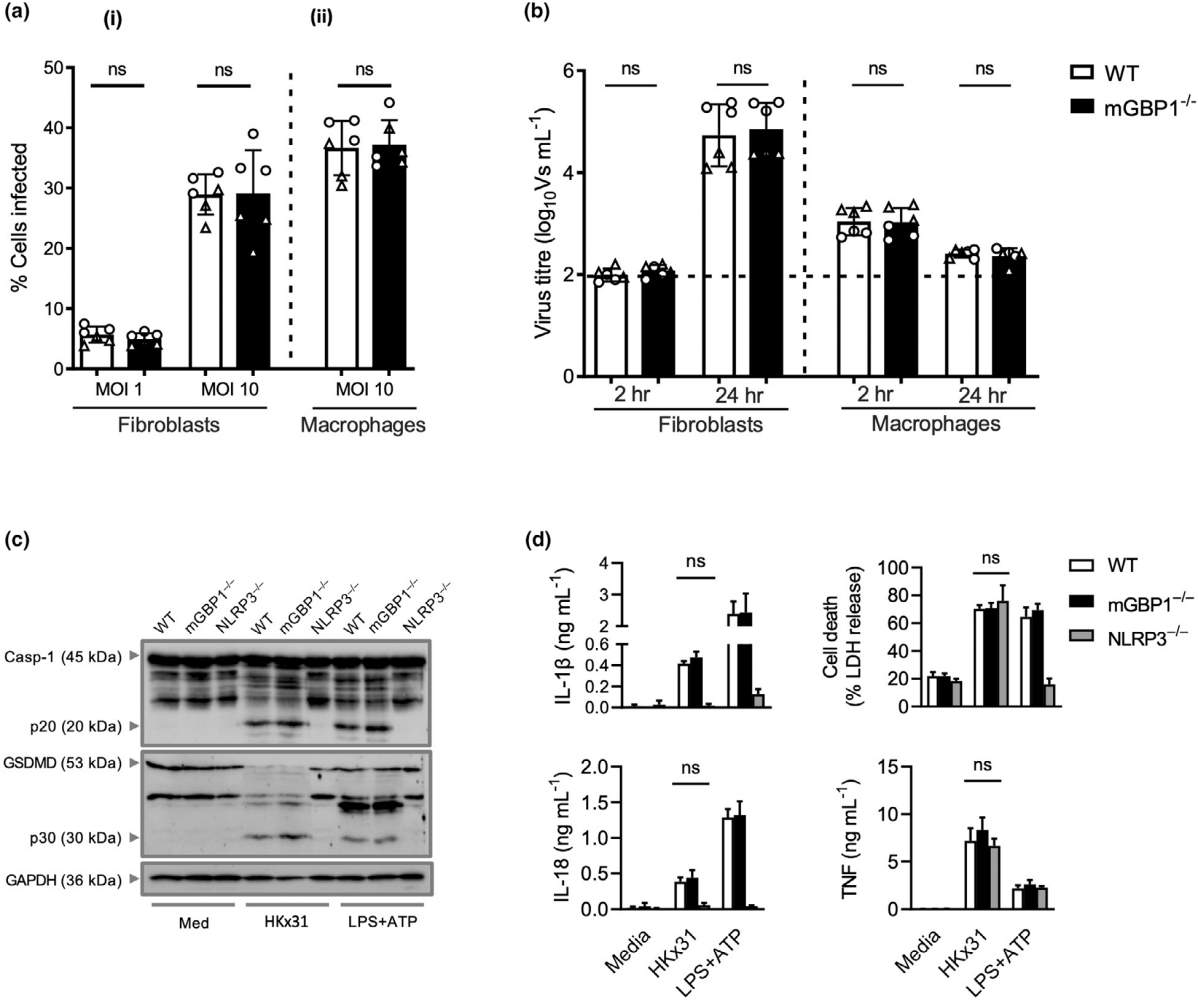
Endogenous mGBP1 does not inhibit IAV infection and replication in primary lung fibroblasts and peritoneal macrophages or IAV‐induced inflammasome activation in BMDMs *in vitro*. Lung primary fibroblasts (2 × 10^5^ cells/well) and peritoneal macrophages (10^5^ cells/well) from WT and mGBP1^−/−^ mice were seeded into 12‐well plates or 8‐well chamber slides, respectively, and incubated overnight at 37°C. The next day cells were infected with the IAV strain HKx31 at the indicated MOI. **(a)** At 8 h p.i. (1) fibroblasts were detached, stained for intracellular viral NP and analyzed by flow cytometry; and (2) peritoneal macrophages were fixed, stained for intracellular viral NP and counted under a fluorescent microscope to determine the percent of infected cells. **(b)** At 24 h p.i., supernatants were removed, clarified and titers of infectious virus were determined by the VS assay on MDCK cells. Data for **a** and **b** show the mean ± standard deviation of triplicate samples pooled from two independent experiments, represented as circles and triangles, respectively. The dashed line represents the detection limit of the VS assay. Statistical significance was determined by the Student's *t*‐test. **(c)** Immunoblot analysis of pro‐casapse‐1 (Casp‐1) and the active caspase‐1 p20 subunit, pro‐pyroptosis effector protein GSDMD, the active GSDMD p30 subunit of WT, and NLPR3^−/−^ and mGBP1^−/−^ BMDMs left untreated or infected with IAV for 16 h. Blots shown have been cropped for presentation. **(d)** Levels of IL‐1β, IL‐18, LDH and TNF in cell culture supernatants 16 h after infection with IAV. Data show the mean ± standard deviation of three independent experiments. BMDM, bone marrow–derived macrophage; GAPDH, glyceraldehyde 3‐phosphate dehydrogenase; GSDMD, gasdermin D; IAV, influenza A virus; IL, interleukin; LDH, lactate dehydrogenase; LPS, lipopolysaccharide; MDCK, Madin–Darby Canine Kidney; mGBP1, murine guanylate‐binding protein 1; MOI, multiplicity of infection; NLRP3, NLR family pyrin domain containing 3; NP, nucleoprotein; ns, not significant; p.i., postinfection; TNF, tumor necrosis factor; VS, ViroSpot; WT, wild type.

The activity of ectopically overexpressed mGBP1 may not be representative of endogenous mGBP activity, because protein overexpression can be associated with protein aggregation, overload of specific biological pathways and/or disruption of cellular regulation (reviewed in Bolognesi and Lehner^25^). To determine whether endogenous mGBP1 could modulate IAV infection and/or replication *in vitro*, primary lung fibroblasts and peritoneal macrophages from C57BL/6Ncrl [wild type (WT)] and mGBP1^−/−^ mice^26^ were examined for susceptibility to IAV infection and ability to support IAV replication. As seen in Figure 4a, each cell population derived from WT or mGBP1^−/−^ mice was equally susceptible to the early stages of IAV infection. Moreover, lung fibroblasts from WT or mGBP1^−/−^ mice did not show any difference in their ability to support IAV replication, as seen by a similar increase in the titer of virus released from IAV‐infected cells between 2 and 24 h after infection (Figure 4b). We and others have reported that mouse macrophages restrict productive replication of seasonal IAV (reviewed in Meischel *et al*.^27^). Consistent with this, we saw no increase in virus titers released from WT peritoneal macrophages between 2 and 24 h after infection and this was not altered in the absence of endogenous mGBP1 (Figure 4b).

IAV infection of mouse bone marrow–derived macrophages (BMDMs) triggers activation of the NLRP3 (NLR family pyrin domain containing 3) inflammasome that is critical for virus inhibition.^28^ Several components of IAV contribute to inflammasome activation including viral RNA,^29^ the viral M2 protein^28^ and the viral PB1‐F2 peptide.^30^, ^31^ Indeed, mGBPs on chromosome 3 have been shown to release concealed ligands from intracellular bacteria to facilitate inflammasome activation (reviewed in Ngo and Man^32^); however, it is unknown whether GBPs are important in inflammasome activation in response to IAV infection. To determine whether mGBP1 could modulate IAV‐induced inflammasome activation, we infected primary WT, NLRP3^−/−^ and GBP1^−/−^ BMDMs with IAV [multiplicity of infection (MOI) 25] for 16 h. Note that this high MOI is typical of studies examining inflammasome activation during IAV infections.^33^ IAV infection induced caspase‐1 activation, cleavage of gasdermin D and secretion of interleukin (IL)‐1β and IL‐18 in both WT and GBP1^−/−^ BMDMs, but not in NLRP3^−/−^ BMDMs (Figure 4c, d). In comparison, lactate dehydrogenase release was unaltered between IAV‐infected WT, GBP1^−/−^ and NLRP3^−/−^ BMDMs. Further, GBP1 was not required for activation of the NLRP3 inflammasome by the canonical NLRP3 activator lipopolysaccharide plus adenosine triphosphate (ATP) (Figure 4c, d). Collectively, these data indicate that mGBP1 is dispensable for IAV‐mediated activation of the NLRP3 inflammasome.

### Mice lacking mGBP1 show no major differences in susceptibility to IAV infection

**Figure 5:**
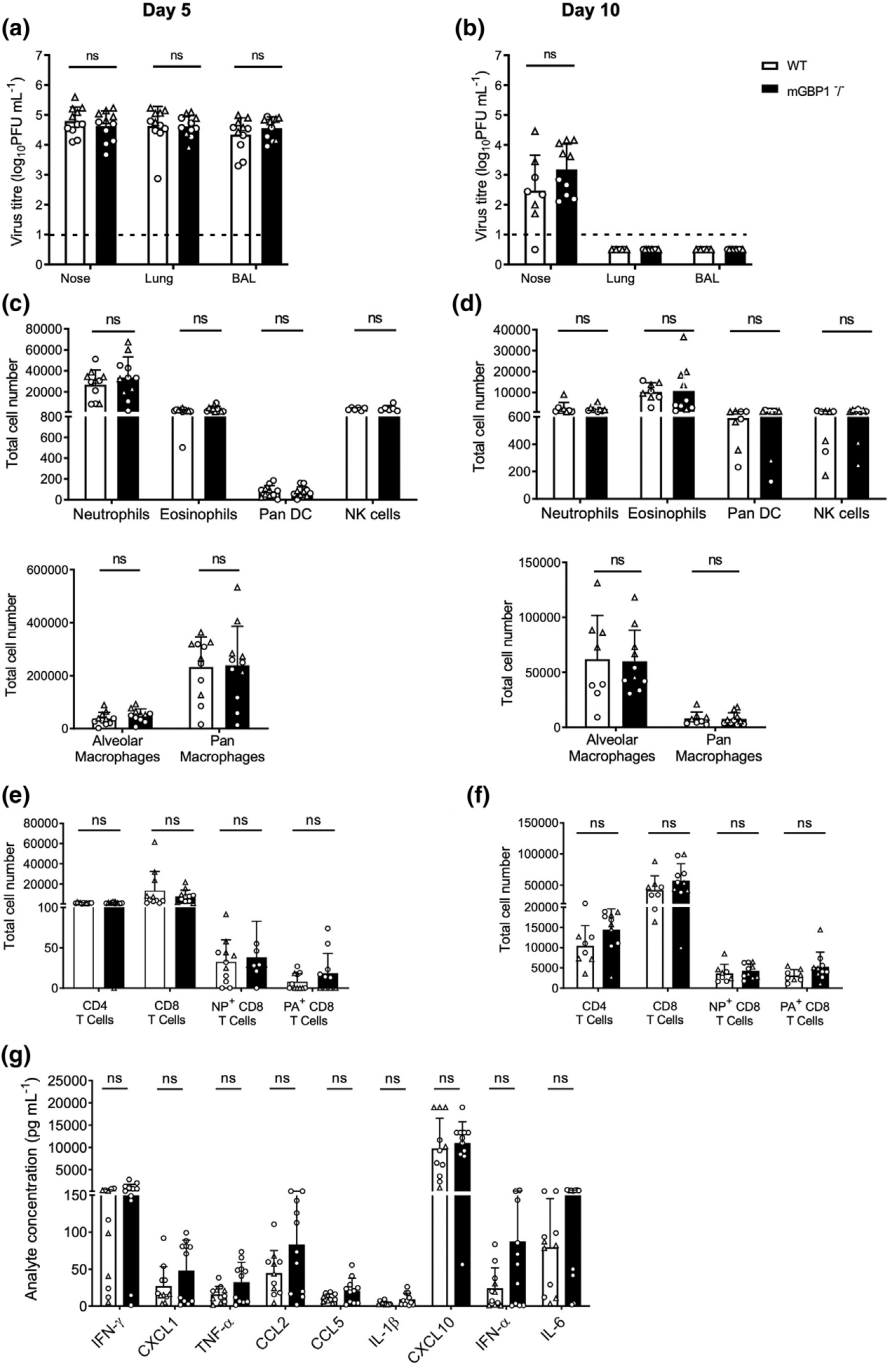
Titers of infectious virus and inflammatory responses detected from WT and mGBP1^−/−^ mice infected with HKx31. Mice were infected *via* the intranasal route with 50 μL of PBS containing 10^3.5^ PFU of the IAV strain HKx31. At days 5 **(a, c, e, g)** or 10 **(b, d, f)** p.i., mice were killed and BAL was performed, before removal of lungs and nasal tissues. **(a, b)** Virus titers in homogenates prepared from nasal tissues or lungs, or in BAL, were determined by the plaque assay using MDCK cells. The broken line indicates the limit of detection of the plaque assay. **(c–f)** BAL cells were stained with antibodies against specific markers and flow cytometry was used to identify and quantify populations of **(c, e)** innate immune cells and **(d, f)** T cells. **(g)** Cell‐free BAL fluid was analyzed by flow cytometry using a multiplex CBA assay to detect 13 different inflammatory cytokines and chemokines. All data **(a–f)** show mean ± standard deviation values from mice pooled from two independent experiments, represented as circles and triangles, respectively (*n* = 8 or 11/group in total). A two‐tailed unpaired Student's *t*‐test was performed to compare viral titers, numbers of immune cell populations and cytokines and chemokines released between WT and mGBP1^−/−^ mice. No significant differences were noted between WT and mGBP1^−/−^ mice. BAL, bronchoalveolar lavage; CBA, cytometric bead array; CCL, chemokine (C–C motif) ligand; CXCL, C–X–C motif chemokine ligand; DC, dendritic cell; IAV, influenza A virus; IFN, interferon; IL, interleukin; MDCK, Madin–Darby Canine Kidney; mGBP1, murine guanylate‐binding protein 1; NP, nucleoprotein; ns, not significant; PA, polymerase acidic; PBS, phosphate‐buffered saline; PFU, plaque‐forming unit; p.i., postinfection; TNF, tumor necrosis factor; WT, wild type.

*In vitro* studies indicate that neither DOX‐inducible or endogenous mGBP1 modulate IAV infection, nor did endogenous mGBP1 play a critical role in IAV‐induced inflammasome activation in BMDM. However, as mGBP1 has been implicated in modulating cell proliferation and apoptosis,^34^ as well as inflammatory responses during viral infection,^35^ it was important to assess its impact during IAV infection *in vivo*. Therefore, WT and mGBP1^−/−^ mice were infected *via* the intranasal route with 10^3.5^ plaque‐forming units (PFU) of the IAV strain HKx31 to allow for assessment of virus replication and inflammation at early (day 5) and later (day 10) timepoints. At days 5 and 10 after infection, mice were killed and virus titers determined in homogenates prepared from the nose and lungs, or from bronchoalveolar lavage (BAL). As seen in Figure 5a, high titers of virus were present in nasal tissues and lungs at day 5 after infection; however, no significant differences were noted between samples from WT or mGBP1^−/−^ mice. By day 10 after infection, virus had been cleared from BAL and lungs of all mice and no differences were noted in the low virus titers present in nasal tissues from WT or mGBP1^−/−^ mice (Figure 5b).

Next, we used flow cytometry to determine whether the absence of mGBP1 altered numbers of inflammatory cells in the airways at day 5 or day 10 after infection. Cell populations were broadly classified into innate cell populations (including natural killer cells, neutrophils, eosinophils, alveolar macrophages, pan‐macrophages and pan‐DC) and T cells (including CD4^+^ and CD8^+^ T cells, as well as polymerase acidic (PA)‐ and nucleoprotein (NP)‐specific CD8^+^ T cells), as described in the “Methods” section. BAL from naïve mice contains very low cell numbers (5 × 10^4^ cells/mouse) of which over 90% are alveolar macrophages (data not shown). At day 5 after infection, total BAL cell numbers had markedly increased for both WT (3.2 × 10^5^ ± 1.5 × 10^5^ cells/BAL; *n* = 11) and mGBP1^−/−^ mice (3.4 × 10^5^ ± 1.8 × 10^5^ cells/BAL; *n* = 11), with the majority of infiltrating cells being pan‐macrophages, alveolar macrophages and neutrophils (Figure 5c). At day 10 (when virus had been cleared from the lungs), overall cellularity of the BAL was markedly reduced, and alveolar macrophages predominated over other innate cell populations (Figure 5d). No statistically significant differences were noted in any innate cell population between WT and mGBP1^−/−^ mice at either timepoint. At day 5 after infection, low numbers of CD4^+^ and CD8^+^ T cells were detected in BAL (Figure 5e) and these increased markedly, particularly for CD8^+^ T cells, by day 10 after infection (Figure 5f). No significant differences were noted in the numbers of CD4^+^ or CD8^+^ T cells between WT and mGBP1^−/−^ mice at either timepoint, including numbers of PA‐ and NP‐specific CD8^+^ T cells infiltrating the lung at day 10 after infection.

Finally, we used a multiplex cytometric bead array assay to determine levels of inflammatory cytokines and chemokines in cell‐free BAL fluid at days 5 and 10 after infection. Analysis of individual mediators indicated that day 5 responses in BAL were dominated by CXCL10 (C–X–C motif chemokine ligand 10) and, to a lesser extent, by IFNγ, CXCL1, CCL2 [chemokine (C–C motif) ligand 2], CCL5, tumor necrosis factor‐α, IFNα, IL‐1β and IL‐6 (Figure 5g) with levels of granulocyte–macrophage colony‐stimulating factor, IL‐12, IL‐10 and IFNβ below detection. When comparing each mediator from WT or mGBP1^−/−^ mice, no significant differences were noted for any chemokine or cytokine tested. At day 10 after infection, all inflammatory mediators had decreased to levels below 10 pg mL^−1^, except for CXCL10, which was detected in BAL from both WT (38.2 ± 55.1 pg mL^−1^; *n* = 8) and mGBP1^−/−^ mice (38 ± 52.6 pg mL^−1^; *n* = 10). Again, no significant differences were noted between WT and mGBP1^−/−^ mice in levels of particular inflammatory mediators at day 10 after infection.

## DISCUSSION

In contrast to emerging literature defining the ability of hGBPs to inhibit different viruses *in vitro*, studies describing the antiviral properties of mGBPs, especially mGBP1, are limited. Ectopic expression of mGBP2 has been reported to inhibit VSV and encephalomyocarditis virus^36^ and mGBP2 was recently shown to mediate IFNγ‐dependent antiviral activity against murine norovirus‐1 in mouse macrophages, noting that the viral NS2 protein colocalized with and antagonized its function.^37^ As we demonstrated upregulation of mGBP1 mRNA in response to IAV infection *in vitro*, we therefore investigated its potential to mediate anti‐IAV activity. LA‐4 cells with DOX‐inducible expression of mGBP1 (or mMx1, a related GTPase protein) were generated, with mMx1 acting as a control protein known to mediate potent anti‐IAV activity. In our experimental system, inducible expression of FLAG‐tagged mMx1 potently inhibited IAV infection and growth, whereas FLAG‐tagged mGBP1 did not. The DOX‐inducible expression system has a number of advantages and allows for controlled expression of mGBP1, including upregulating expression prior to IAV infection (as performed in our studies) to maximize its potential impact on virus replication. However, as DOX has been reported to impact inflammatory cytokine secretion,^38^ as well cell metabolism and proliferation,^39^ this limits its utility to examine other aspects of virus infection, such as the induction of inflammatory mediators. It might also be possible that the N‐terminal FLAG tag inhibited the anti‐IAV activity of mGBP1, although this tag did not impair the ability of mMx1 to inhibit the same viruses.^22^ These limitations highlight the importance of complementary *in vitro* and *in vivo* studies using mGBP1^−/−^ mice.

*In vitro* studies assessed the impact of endogenous mGBP1 on IAV infection using primary lung fibroblasts or macrophages from mGBP1^−/−^ mice. Here, we observed no difference in IAV infection or growth in cells from WT *versus* mGBP1^−/−^ mice. Using the same mice for *in vivo* studies, we report that mGBP1^−/−^ mice showed no difference in virus replication or inflammatory responses elicited in the airways following IAV infection. In a recent report, we demonstrated impaired inflammasome activation in BMDM from the same mGBP1^−/−^ mice in response to the cytosolic bacterium *Francisella novicida*, which correlated with enhanced intracellular replication in BMDM.^26^ Moreover, mGBP1^−/−^ mice showed increased susceptibility to infection and harbored more viable *F. novicida* in the liver and spleen compared with WT mice.^26^ While mGBP1^−/−^ mice clearly represent a useful tool to define the importance of mGBP1 against certain pathogens, data presented herein indicate that endogenous mGBP1 is not a major modulator of IAV infection *in vitro* or *in vivo*. In an earlier study, Pan *et al*.^19^ demonstrated that *in vitro* silencing of endogenous mGBP1 was associated with increased in titers of dengue virus (New Guinea C strain) released from RAW264.7 macrophages (from <5 plaques to ~15 plaques at 24 h after infection). Aside from this very modest effect, we are not aware of other studies demonstrating the ability of mGBP1 to inhibit any other viruses *in vitro*.

While our studies indicate that mGBP1 is not directly antiviral against IAV, mGBP2 was recently shown to mediate IFNγ‐dependent antiviral activity against murine norovirus‐1 in mouse macrophages, noting that the viral NS2 protein colocalized with and antagonized its function.^37^ Overexpression studies have also demonstrated that mGBP2 can inhibit VSV and encephalomyocarditis virus replication and that the GTP‐binding motif was necessary for mGBP2‐mediated inhibition of encephalomyocarditis virus, but not VSV.^36^ In terms of IAV, a number of specific host restriction factors have been shown to inhibit replication at different stages of the virus replication cycle, including during virus entry and uncoating [e.g. interferon‐induced transmembrane protein (IFITM)3], viral genomic replication (e.g. mMx1) and assembly and budding (e.g. tetherin; reviewed in Villalon‐Letelier *et al*.^40^). Among these, several host proteins that localize to the cytoplasm have been reported to antagonize IAV in different ways, including hMxA which oligomerizes to form stable complexes with NP in the cytoplasm at a replication step following primary transcription.^41^ For Mx proteins, it is also well established that a functional GTP‐binding domain is critical for the potent anti‐IAV activity.^42^ Like hMxA, mGBP1 is also expressed in the cytoplasm and encodes a functional GTP‐binding domain; however, other features of the mGBP1 protein and/or its specific cytoplasmic localization do not appear sufficient for it to mediate anti‐IAV activity similar to that of hMxA. Given its cytoplasmic localization, we also tested the ability of DOX‐inducible mGBP1 to inhibit productive replication by paramyxoviruses, which replicate exclusively in cytoplasmic compartments. However, we did not detect any differences in virus titers recovered from untreated or DOX‐treated cells infected with human parainfluenza virus‐3 or murine Sendai virus (data not shown).

It is interesting to note that most publications describing GBP‐mediated antiviral activities have focused on human, rather than mouse, GBPs. Studies reporting anti‐IAV activity for hGBP1 have used transfection‐based overexpression approaches to demonstrate dose‐dependent inhibition of virus growth, as well as interactions with the IAV NS1 protein which inhibit its anti‐IAV activity.^15^ Therefore, while others report that hGBP1 inhibits IAV *in vitro* we find that mGBP1 does not. These findings are not without precedent because hGBP1 and mGBP1 clearly differ in biological functions. For example, hGBP1 binds to lipopolysaccharide from *Salmonella typhimurium*, *Shigella flexneri* and other Gram‐negative bacteria, resulting in recruitment of additional hGBPs and subsequent inflammasome activation.^43^ By contrast, we recently demonstrated mGBP1 was not required for the recruitment of mGBP2, mGBP3 or mGBP5 to the intracellular bacterium *F. novicida*, but was required to mediate pathogen‐selective inflammasome activation and bacteriolysis.^26^ Moreover, BMDM from mGBP1^−/−^ mice did not show any defect in inflammasome activation in response to *S*. *typhimurium*, *E. coli* or cytosolic lipopolysaccharide.^26^ While the biological functions of human and mouse GBP1 may differ, mGBP1 is clearly required for inflammasome activation during some bacterial infections. By contrast, BMDMs isolated from mGBP1^−/−^ mice showed no defect in IAV‐induced NLRP3 inflammasome activation. In future studies, it will be of interest to compare the impact of other individual mGBPs, across both the chromosome 3 and chromosome 5 mGBP clusters, to determine whether one or more mGBPs might modulate IAV‐induced inflammasome activation.

In summary, we have assessed the impact of mGBP1 overexpression, as well as the presence or absence of endogenous mGBP1, on both susceptibility of cells to IAV infection and their ability to support IAV replication *in vitro*. In addition, we have investigated the role of endogenous mGBP1 in modulating IAV infection *in vivo*. In contrast to its ability to modulate infection by a different intracellular bacterium and protozoon, we report no evidence that mGBP1 mediates antiviral activity against IAV. However, it is possible that one or more mGBPs do modulate IAV infection and that in the absence of mGBP1, other mGBPs with complementary function may mask its impact. For example, during *T. gondii* infection the loss of mGBP5 was compensated for by other mGBPs, including mGBP1 and mGBP7.^44^ It is also possible that antagonism of mGBP1 function by viral proteins could mask any antiviral effects against IAV, as the viral NS1 protein has been reported to antagonize the antiviral activity of hGBP1.^15^ In future studies, it will be of interest to assess cells and mice lacking clusters of mGBPs for their susceptibility to IAV and other viral infections in an effort to gain a better understanding of the ability of mGBPs to modulate viral infections *in vitro* and *in vivo*.

## METHODS

### Cell lines and viruses

Murine lung epithelial (LA‐4) cells (American Type Culture Collection, Manassas, VA, USA) were grown in Ham's F‐12 K (Kaighn's) medium (Gibco‐BRL, Grand Island, NY, USA) containing 10% (vol/vol) fetal calf serum (FCS; Sigma‐Aldrich, Burlington, MA, USA) and supplemented with 2 mm l‐glutamine, 100 U mL^−1^ penicillin and 10 μg mL^−1^ of streptomycin (Gibco‐BRL). Madin–Darby Canine Kidney cells (American Type Culture Collection) and the human kidney epithelial cell line 293 T (American Type Culture Collection) were cultured in RPMI (Roswell Park Memorial Institute) medium (Gibco‐BRL) and Dulbecco's modified Eagle medium (Gibco‐BRL), respectively, supplemented with 10% (vol/vol) FCS and additives as above. The IAV strains used in this study were A/PR/8/34 (PR8, H1N1), A/Tasmania/2004/09 (Tas/09, H1N1pdm09), A/HKx31 (HKx31, H3N2), A/Phil/82X (Phil/82, H3N2) and A/Sydney/203/2000 (H3N2). HKx31 and Phil/82x are both high‐yielding reassortants of A/Aichi/2/68 (H3N2) or A/Philippines/2/82 (H3N2) with PR8, respectively. Each reassortant virus bears the surface glycoproteins of the H3N2 parent virus. All viruses were grown in embryonated chicken eggs by standard procedures and titers of virus stocks determined by the standard plaque assay on Madin–Darby Canine Kidney cells and expressed in PFU mL^–1^.

### Generating LA‐4 cell lines with doxycycline‐inducible expression of intracellular proteins

To produce lentiviruses for the generation of cell lines with DOX‐inducible expression of GPB and Mx proteins, a two‐step cloning strategy was required. mGBP1 (Accession number NM_010259.2) and mMx1 (Accession number NM_010846.1) engineered to express an N‐terminal FLAG‐tag were cloned into the pTRE‐tight plasmid vector, and then subcloned into the pFUV1‐mCherry lentivirus transfer plasmid^45^ (kindly provided by Associate Professor Marco Herold, The Walter and Eliza Hall Institute, Parkville, Australia). A cell line expressing cytoplasmic hen egg ovalbumin lacking the sequence for cell surface trafficking was prepared as a control. Lentivirus stocks to be used for subsequent LA‐4 cell transduction were generated in 293 T cells following transfection of (1) pMDL (gag and pol), (2) pRSV‐REV packaging plasmids, (3) pMD2G.VSVg envelope plasmid and (4) pFUV1‐mCherry transfer plasmid by standard procedures. Lentivirally transduced LA‐4 cells were sorted based on mCherry‐positive cells using a FACSAria III instrument (BD Biosciences, Franklin Lakes, NJ, USA).

### Detection of FLAG‐tagged proteins in LA‐4 cell lines

#### Flow cytometry

FLAG‐tagged mGBP1 and mMx1 proteins were detected in LA‐4 cells (cultured for 24 h in the presence or absence of 1 μg mL^−1^ DOX) by intracellular staining with allophycocyanin‐conjugated anti‐FLAG monoclonal antibody (mAb; clone L5; BioLegend, San Diego, CA, USA) in conjunction with the fixable viability dye eFluor 780 (eBioscience/Thermo Fisher Scientific, Waltham, MA, USA) before flow cytometric analysis.

#### Western blot

FLAG‐tagged mGBP1 and mMx1 proteins were detected in LA‐4 cells (cultured for 24 h in the presence or absence of 1 μg mL^−1^ DOX) by western blot as described previously,^46^ using an mAb directed to FLAG (clone M2, Sigma‐Aldrich, USA) in conjunction with horseradish peroxidase–conjugated rabbit anti‐mouse antibody (P0260; Dako, Glostrup, Denmark). Cellular β‐actin was monitored to ensure equivalent protein loading of all samples using a mouse mAb (clone sc‐47 778; Santa Cruz, CA, USA). Bound antibodies were detected using enhanced chemiluminescence (Western Lightning plus ECL; Perkin‐Elmer, Shelton, CT, USA) and visualized using an Amersham Imager 600 (GE Healthcare, Chicago, IL, USA) in conjunction with FIJI ImageJ software.

#### Confocal microscopy

FLAG‐tagged mGBP1 and mMx1 proteins were visualized in LA‐4 cells by confocal microscopy. Cells were seeded into 8‐well chamber slides (Lab‐Tek, Nunc, St Louis, MO, USA) (cultured for 24 h in the presence or absence of 1 μg mL^−1^ DOX), and cell surface staining was performed prior to fixation using biotinylated Maackia Amurensis Lectin II (MAL II; Vector Laboratories, Newark, CA, USA) followed by Alexa Fluor 488 streptavidin (Molecular Probes, Eugene, OR, USA) in cold 0.05 m Tris–HCL, 0.15 m NaCl buffer, pH 7.2 containing, 10 mm CaCl_2_ and 1% BSA. Cells were then fixed in phosphate‐buffered saline (PBS) containing 4% (vol/vol) paraformaldehyde before permeabilization in PBS containing 5% (wt/vol) bovine serum albumin, 5% (vol/vol) FCS and 0.1% (vol/vol) Triton X‐100. FLAG was detected using an anti‐FLAG antibody conjugated to Alexa Fluor 647 (L5; BioLegend) followed by additional amplification of the signal using goat anti‐rat immunoglobulin conjugated to Alexa Fluor 647 (Invitrogen, Waltham, MA, USA). Prior to visualization, cells were counterstained with VECTASHIELD Antifade Mounting Medium containing 4′,6‐diamidino‐2‐phenylindole (Vector, Burlingame, CA, USA). Images were acquired with a Zeiss LSM780 microscope and were processed using FIJI ImageJ software (Version 2.1.0/1.53c, National Institutes of Health, Bethesda, MA, USA).

#### Quantitative RT‐PCR for detection of mGBP1 messenger RNA in LA‐4 cells exposed to type I IFN or IAV

LA‐4 cells seeded in 12‐well tissue culture plates (Corning Inc., Corning, NY, USA) were cultured overnight and then treated with 1000 U mL^−1^ of recombinant mouse IFNα (Miltenyi Biotec, Bergisch Gladbach, Germany) or exposed to IAV (HKx31) at an MOI of 5. Mock‐treated cells were included for comparison. RNA was extracted 2, 6, 12 and 24 h after treatment using the RNeasy Mini Kit (Qiagen, Hilden, Germany) according to manufacturer's instructions. RNA purity and quantity were determined by nanodrop (Thermo Fisher Scientific). Total RNA was reverse transcribed into complementary DNA using SensiFAST cDNA Synthesis Kit (Bioline, London, UK). SensiFAST SYBR Lo‐ROX kit (Bioline) was used with primers for mGBP1 (forward: 5′‐ACT TCA GGA ACA GGA AAG ACT TC‐3′; reverse: 5′‐CGA GGT GGA GGC ATG TTC TT‐3′) and mouse glyceraldehyde 3‐phosphate dehydrogenase (forward: 5′‐CCA GGT TGT CTC CTG CGA CTT‐3′; reverse: 5′‐CCT GTT GCT GTA GCC GTA TTCA‐3′) and data were analyzed with a QuantStudio 7 Flex RT‐PCR System (Applied Biosystems, Waltham, MA, USA). Cycling conditions included a first denaturation step (95°C for 2 min), followed by 40 cycles of denaturation (95°C for 5 s), annealing (60°C for 10 s) and extension (72°C for 20 s). Relative gene expression of mGBP1 was normalized to mouse glyceraldehyde 3‐phosphate dehydrogenase and then expressed as a fold change from mock. Relative expressions for each sample were determined using the 2^−ΔΔCT^ method.^47^

#### Virus infection of LA‐4 cells

LA‐4 cells were infected with IAV and the percentage of IAV‐infected cells at 8 h after infection was determined by flow cytometric analysis for IAV NP expression. In brief, cells were incubated with IAV in serum‐free media for 1 h at 37°C. Virus inoculum was removed, and the cells washed and incubated for a further 7 h at 37°C in serum‐free media. IAV‐infected cells were stained‐fixed with 4% paraformaldehyde, and permeabilized with 1% (vol/vol) Triton‐X (Sigma‐Aldrich) and stained in PBS containing 0.5% (vol/vol) Triton‐X and 1% FCS using a fluorescein isothiocyanate–conjugated mAb specific for IAV NP (ab20921; Abcam, Cambridge, UK).

To determine whether infection resulted in amplification and release of infectious virus from target cells, cell monolayers were infected as described above. Cell supernatants were collected at 24 h after infection, clarified by centrifugation and incubated with tosylsulfonyl phenylalanyl chloromethyl ketone (TPCK)–treated trypsin (2 μg mL^−1^; Sigma) for 30 min at 37°C to facilitate cleavage of viral HA_0_, before the titers of infectious virus were determined by the ViroSpot assay. For the ViroSpot assay, Madin–Darby Canine Kidney cells were seeded into 96‐well tissue culture plates (Corning, NY, USA) overnight, washed with serum‐free media and incubated with 10‐fold dilutions of clarified supernatants from virus‐infected cells. After 2 h at 37°C, cells received 100 μL of overlay media (equal volumes of 6.4% carboxymethylcellulose sodium salt (Sigma‐Aldrich) and 2× minimal essential media, containing 2 μg mL^−1^ tosylsulfonyl phenylalanyl chloromethyl ketone–treated trypsin (Sigma‐Aldrich). After incubation for 24 h at 37°C, the overlay was removed, and cell monolayers were fixed in cold 80% (vol/vol) acetone in water. For staining, plates were incubated with 200 μL/well blocking solution (5% wt/vol skimmed milk in PBS containing 0.05% vol/vol Tween 20) for more than 30 min, washed in PBS containing 0.05% (vol/vol) Tween and virus‐infected cells were detected using the IAV NP–specific mAb MP3.10 g2.1C7 (WHO Collaborating Centre for Reference and Research on Influenza, Melbourne, Australia) followed by horseradish peroxidase–conjugated rabbit anti‐mouse antibody (Dako). Virus‐infected cells were visualized by incubating plates with KPL TrueBlue Peroxidase Substrate (Life sciences inc., Milford, MA, USA) and scanned with the CTL ImmunoSpot analyzer (CTL, Shaker Heights, OH, USA). Spots (10–150/well) were counted manually using ImageJ Cell Counter. Virus titers are expressed as ViroSpot mL^−1^ of the original sample.

#### Virus infection of primary lung fibroblasts and peritoneal macrophages

C57BL/6Ncrl (WT) and GBP1^−/−^ mice on a C57BL/6Ncrl background were bred and housed in specific pathogen‐free conditions in the Bioresources Facility of the Peter Doherty Institute for Infection and Immunity, Melbourne, Australia. Derivation and validation of GBP1^−/−^ mice at protein and genomic levels have been previously reported.^26^ All research complied with the University of Melbourne's Animal Experimentation Ethics guidelines and policies. Primary lung fibroblasts^48^ and peritoneal macrophages^46^ generated from naïve WT and GBP1^−/−^ mice were infected with the IAV strain HKx31 at the indicated MOI. Cells were assessed at 8 h after infection to determine the percent of infected cells by flow cytometry (fibroblasts) as described above, or by immunofluorescence (macrophages). Supernatants were collected at 24 h after infection to determine titers of infectious virus in clarified supernatants by the ViroSpot assay as described above.

IAV infection of peritoneal macrophages was determined at 8 h after infection by immunofluorescence as described previously,^46^ using the NP‐specific mAb MP3.10 g2.1C7, followed by fluorescein isothiocyanate–conjugated goat anti‐mouse immunoglobulin (Millipore, Burlington, MA, USA). The percentage of infected cells was determined by costaining with 4′,6‐diamidoino‐2‐phenylindole and counting the total number of cells *versus* fluorescein isothiocyanate–positive cells under 100× magnification. A minimum of four random fields were selected for counting, assessing at least 200 cells for each sample.

#### Assessing IAV‐induced inflammasome activation in murine bone marrow–derived macrophages

Primary BMDMs derived from WT, GBP1^−/−^ ^26^ and NLRP3^−/−^ mice^49^ were grown for 5–6 days in Dulbecco's modified Eagle medium supplemented with 10% (vol/vol) FCS (Sigma‐Aldrich), 30% (vol/vol) L929 conditioned media and 100 U mL^−1^ penicillin and streptomycin (Gibco) as described previously.^50^ BMDMs were seeded in antibiotic‐free media in 12‐well plates. To activate the inflammasome, BMDMs were infected with IAV (HKx31; MOI 25) in serum‐free media and supplemented with 10% vol/vol FCS 2 h after infection. To activate the canonical NLRP3 inflammasome as a control, BMDMs were primed with 500 ng mL^−1^ ultrapure lipopolysaccharide from *E. coli* (Enzo Life Sciences, Farmingdale, NY, USA) for 3 h and stimulated with 5 mm adenosine triphosphate (ATP, Roche) for 45 min. Cell culture supernatants and cell lysates were collected for lactate dehydrogenase, cytokine and immunoblotting analyses at 16 h after infection. Levels of lactate dehydrogenase released by cells were determined using a CytoTox 96 Non‐Radioactive Cytotoxicity Assay according to manufacturer's instructions (Promega, Madison, WI, USA). Cytokine levels were determined using a multiplex ELISA kit (Millipore) and an IL‐18 ELISA kit (Thermo Fisher Scientific) according to manufacturer's instructions. Caspase‐1 and gasdermin D protein were detected in BMDM lysates by western blotting using the primary antibodies AG‐20B‐0042, Adipogen and ab209845, Abcam, respectively, in conjunction with horseradish peroxidase–conjugated secondary antibody and Clarity Western ECL substrate (Bio‐Rad, Hercules, CA, USA) and the ChemiDoc Touch Imaging System (Bio‐Rad).

#### Infection and analysis of virus‐infected mice

Infection: Mice aged 6–10 weeks were anesthetized and infected with 10^3.5^ PFU of IAV (HKx31) in 50 μL of PBS *via* the intranasal route. At day 5 or 10 after infection, BAL, lung and nasal tissue samples were collected as described.^51^ The plaque assay was used to determine titers of infectious virus in cell‐free BAL, as well as in clarified homogenates prepared from the lungs and nasal tissues of IAV‐infected mice.

Analysis of cytokine response: The BD cytometric bead array mouse anti‐virus response panel kit (BD Biosciences, San Diego, CA, USA) was used to determine levels of IFNγ, CXCL1, tumor necrosis factor‐α, CCL2, IL‐12, CCL2, IL‐1β, CXCL10, granulocyte–macrophage colony‐stimulating factor, IL‐10, IFNβ, IFNα and IL‐6 in cell‐free BAL, as per manufacturer's instructions, by flow cytometric analysis. Analyte concentration was expressed as pg mL^−1^ ± standard deviation.

Immune cell enumeration and phenotyping: Single‐cell suspensions prepared from the BAL of IAV‐infected mice were treated with Red Blood Cell Lysing Buffer Hybri‐Max (Sigma‐Aldrich) to lyse erythrocytes and incubated on ice for 30 min with supernatants from hybridoma 2.4G2 to block Fc receptors. Cell numbers and viability were assessed *via* trypan blue exclusion using a hemocytometer. Immune cell populations were defined as follows: neutrophils (Ly6G^+^, CD11b^+^), eosinophils (Siglec‐F^+^, CD11b^+^, CD64^−^), natural killer cells (NK1.1^+^, CD3^−^), alveolar macrophages (CD64^+^, Siglec‐F^+^, CD11c^+^), pan‐macrophages (CD64+, Siglec‐F^−^, CD11b^+^), pan‐dendritic cells (pan‐DC, CD64^−^, CD11c^+^, MHC II^+^, CD24^+^), CD4^+^ T cells (CD3^+^_,_ CD4^+^) and CD8^+^ T cells (CD3^+^_,_ CD8^+^). As such, cells were stained with appropriate combinations of fluorescent‐conjugated mAbs to cell surface markers using Pacific Blue anti‐CD45.2, BV711 anti‐TCR‐β (BD Biosciences), PerCP/Cy5.5 anti‐CD3, Alexa Fluor 700 anti‐CD4 (GK1.5; eBioscience), PE‐Cy7 anti‐CD8α (53–6.7), BV605 anti‐NK1.1 (PK136), PE anti‐Ly6G (1A8; BD Biosciences), PE CF594‐anti‐Siglec‐F (E50‐2440; BD Biosciences), APC‐anti‐CD64 (X54‐5/7.1), PE‐Cy7 anti‐CD24 (M1/69), BV605 anti‐CD11b (M1/70), BV785 anti‐CD11c (N418), Alexa Fluor 700 anti‐MHC II (M5/114.15.2) and analyzed by flow cytometry. All mAbs were from BioLegend unless specified. Fixable viability dye eFluor 780 (eBioscience) was added to exclude dead cells. In some experiments, cells were stained with phycoerythrin‐ or allophycocyanin‐labeled DbPA224 (acid polymerase; SSLENFRAYV) or DbNP366 (NP; ASNENMETM)‐specific MHC‐I tetramers before staining with anti‐CD8 mAb.

All research with animals was conducted in accordance with the NHMRC Australian code of practice for the care and use of animals for scientific purposes with approval from the University of Melbourne Biochemistry & Molecular Biology, Dental Science, Medicine, Microbiology and Immunology, and Surgery Animal Ethics Committee (project 10 233; approved on December 18, 2018).

### Statistical analysis

Analyses were performed using Microsoft Excel (version 16.69.1) and GraphPad Prism (version 9.2.0, GraphPad Software, San Diego, CA, USA). Statistical significance was determined by the Student's *t*‐test or by one‐way ANOVA, as indicated. Data are shown as means ± standard deviation.

## AUTHOR CONTRIBUTIONS

**Melkamu Bezie Bezie Tessema:** Data curation; formal analysis; investigation; methodology; writing – original draft; writing – review and editing. **Daniel Enosi Tuipulotu:** Formal analysis; investigation; methodology; writing – review and editing. **Clare Oates:** Investigation; methodology; writing – review and editing. **Andrew Brooks:** Funding acquisition; project administration; resources; supervision; writing – review and editing. **Si Ming Man:** Conceptualization; funding acquisition; project administration; resources; supervision; writing – review and editing. **Sarah Londrigan:** Funding acquisition; project administration; resources; supervision; writing – review and editing. **Patrick Reading:** Conceptualization; funding acquisition; investigation; project administration; supervision; writing – original draft; writing – review and editing.

## CONFLICT OF INTEREST

The authors declare no conflict of interest.

## Supporting information

 

## Data Availability

All data associated with this manuscript are available upon request.
