# Supplementary material for: Mouse guanylate‐binding protein 1 does not mediate antiviral activity against influenza virus in vitro or in vivo
Source: Immunol Cell Biol. 2023 Feb 27;101(5):383–96. doi: 10.1111/imcb.12627 (PMC10952839; doi:10.1111/imcb.12627)
Supplement: Supplementary file 1 [file IMCB-101-383-s001.pdf]

Supplementary information 1

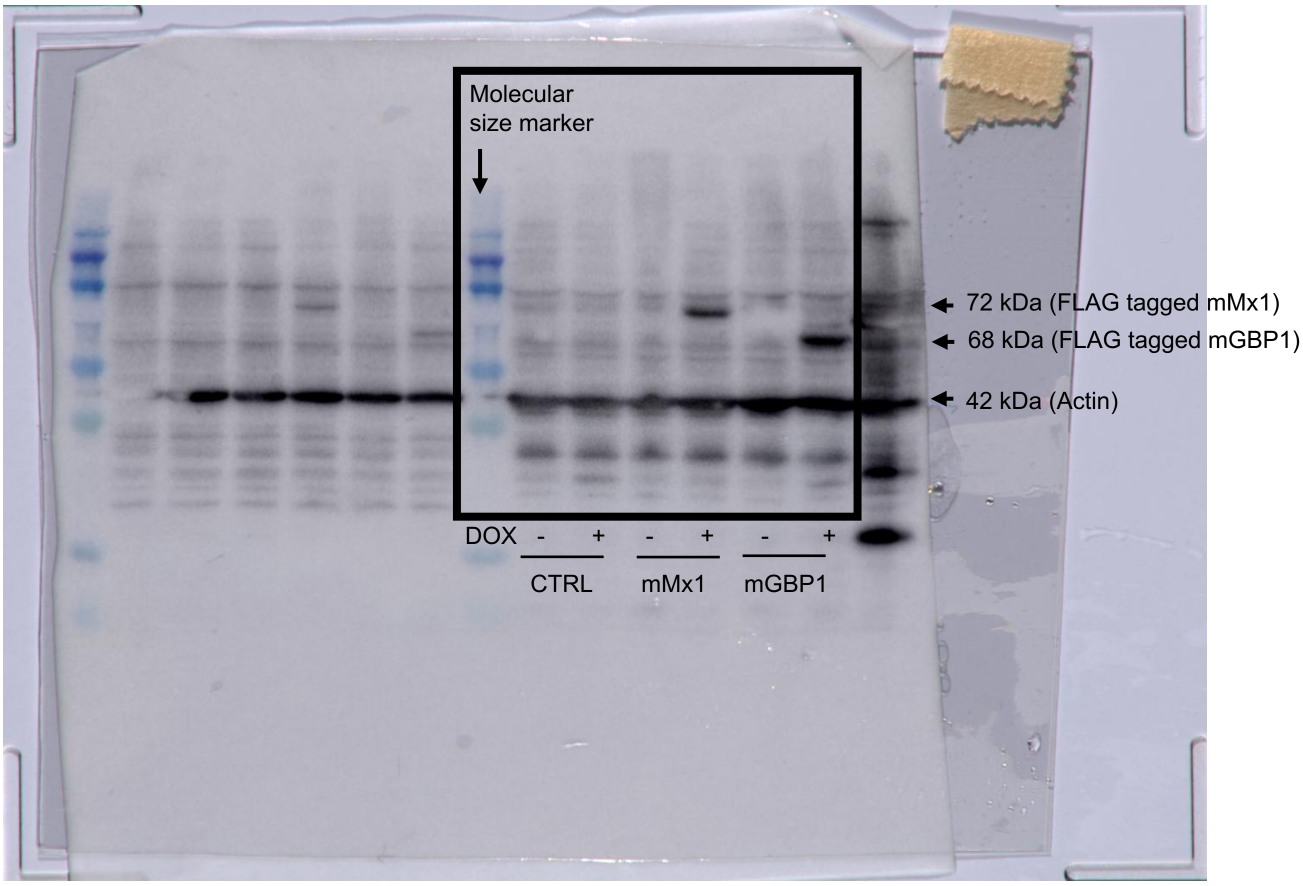

Full-length western blot for figure 2(b)

Supplementary information 2

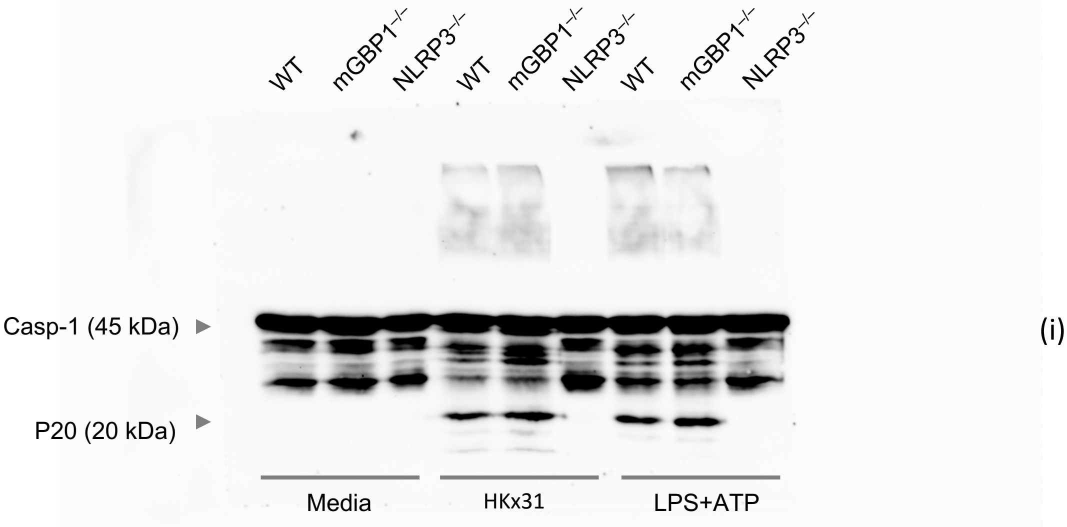

(i)

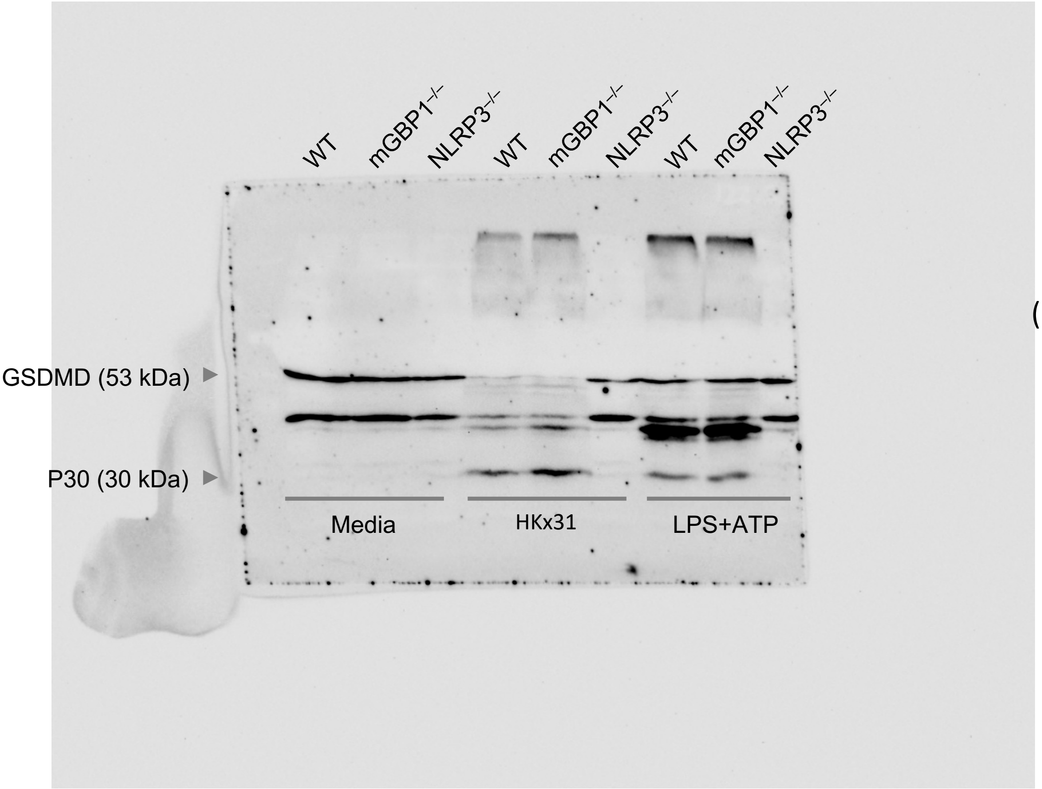

(ii)

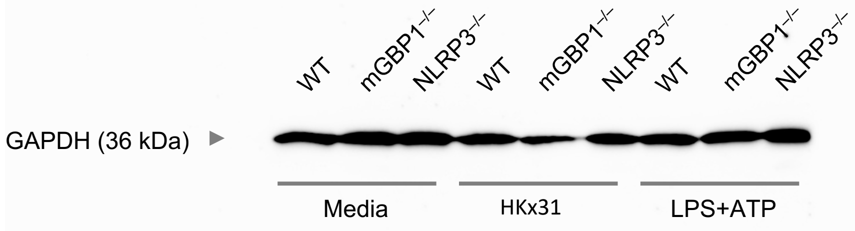

(iii)

Full-length western blot for figure 4(c) – Exposure 1

Supplementary information 2

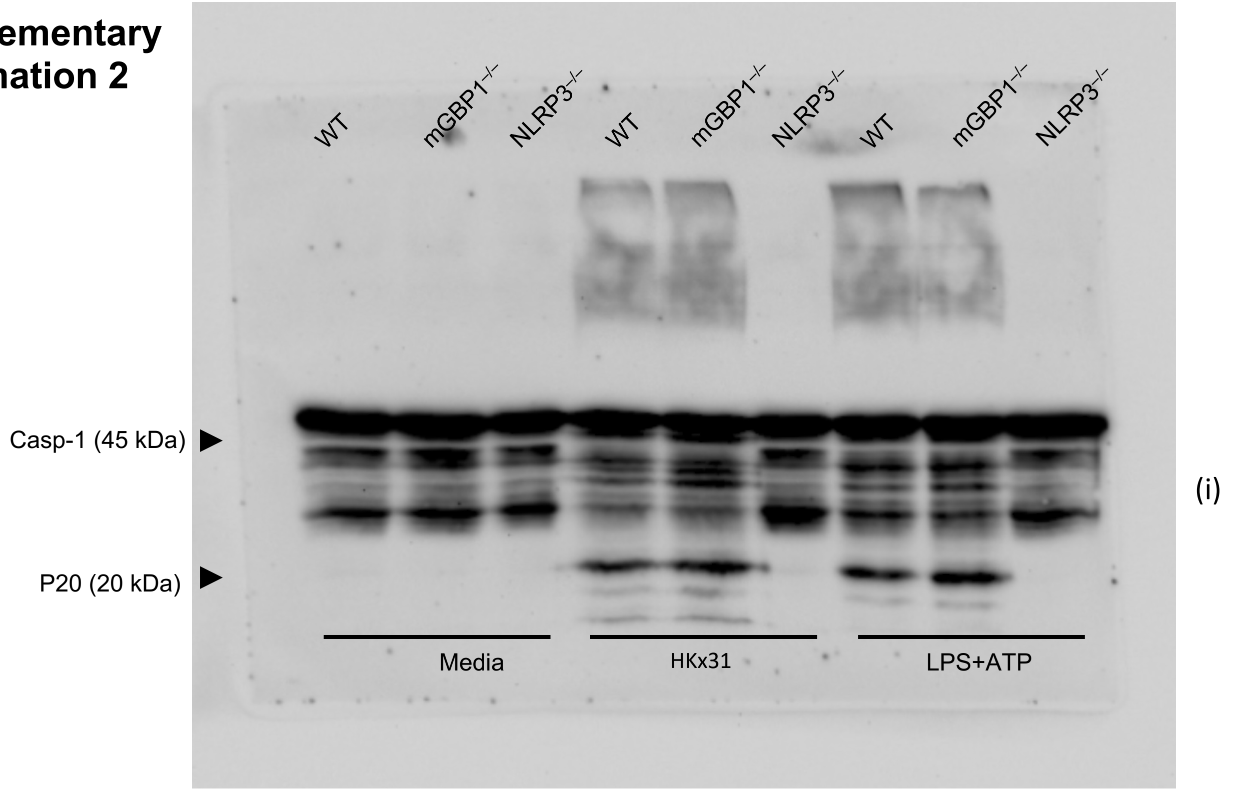

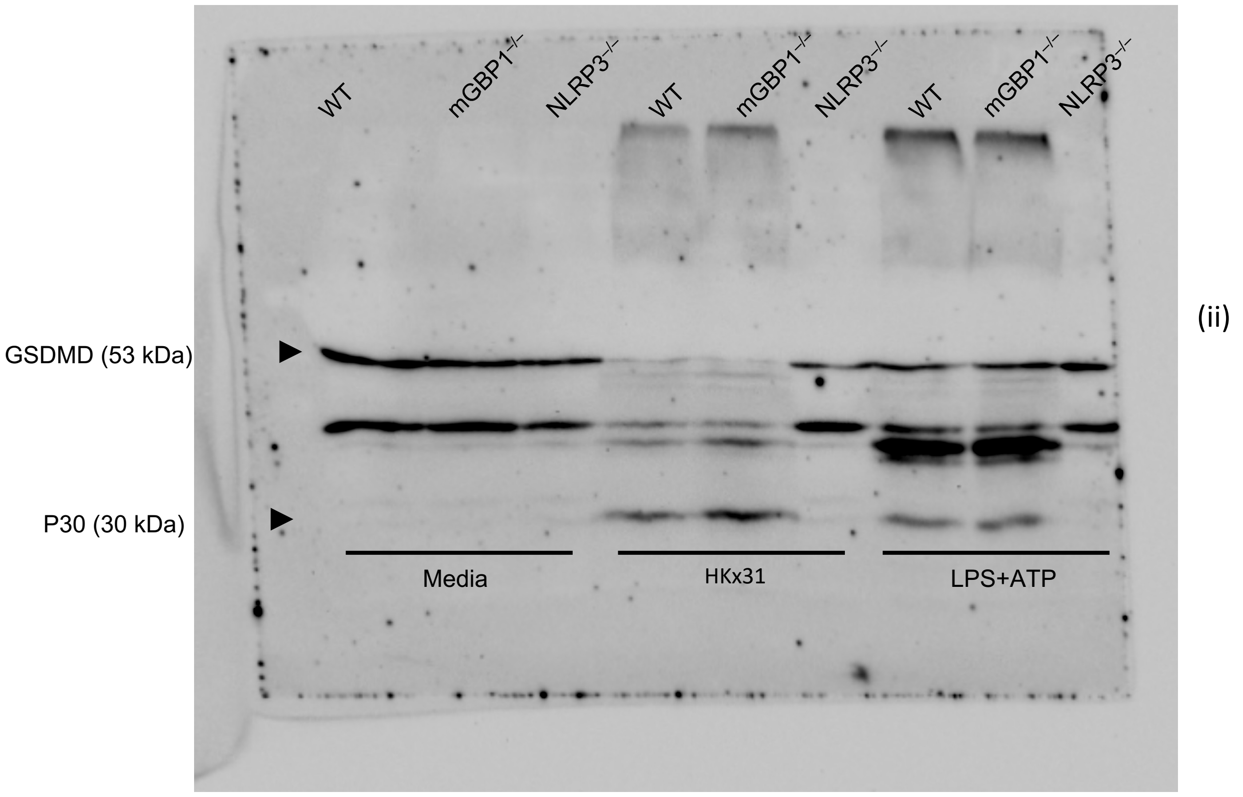

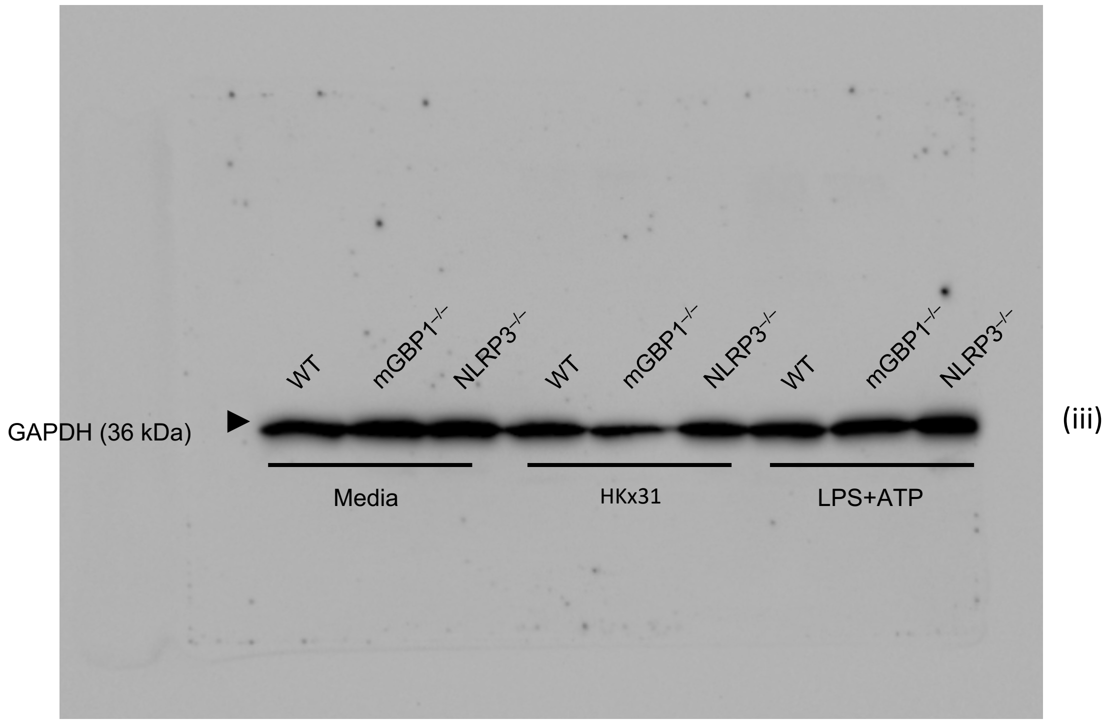

Full-length western blot for figure 4(c) – Exposure 2
